# The Development of an AAV-Based CRISPR SaCas9 Genome Editing System That Can Be Delivered to Neurons *in vivo* and Regulated via Doxycycline and Cre-Recombinase

**DOI:** 10.3389/fnmol.2018.00413

**Published:** 2018-11-13

**Authors:** Namrata Kumar, William Stanford, Christopher de Solis, Aradhana, Nigel D. Abraham, Trieu-Mi J. Dao, Sadiqa Thaseen, Anusha Sairavi, Cuauhtemoc Ulises Gonzalez, Jonathan E. Ploski

**Affiliations:** School of Behavioral and Brain Sciences, Department of Molecular & Cell Biology, The University of Texas at Dallas, Richardson, TX, United States

**Keywords:** AAV vectors, CRISPR/Cas9, SaCas9, Cre recombinase, inducible promoter, doxycycline, neurons, genome editing

## Abstract

The RNA-guided Cas9 nuclease, from the type II prokaryotic clustered regularly interspersed short palindromic repeats (CRISPR) adaptive immune system, has been adapted by scientists to enable site specific genome editing of eukaryotic cells both *in vitro* and *in vivo*. Previously, we reported the development of an adeno-associated virus (AAV)-mediated CRISPR *Streptococcus pyogenes* (Sp) Cas9 system, in which the genome editing function can be regulated by controlling the expression of the guide RNA (sgRNA) in a doxycycline (Dox)-dependent manner. Here, we report the development of an AAV vector tool kit utilizing the Cas9 from *Staphylococcus aureus* (SaCas9). We demonstrate *in vitro* genome editing in human derived 293FT cells and mouse derived Neuro2A (N2A) cells and *in vivo* in neurons of the mouse brain. We also demonstrate the ability to regulate the induction of genome editing temporally with Dox and spatially with Cre-recombinase. The combination of these systems enables AAV-mediated CRISPR/Cas9 genome editing to be regulated both spatially and temporally.

## Introduction

The CRISPR/Cas system has become an extremely useful tool in probing biology in both clinical and scientific settings. Initially, the CRISPR/Cas system was adapted from the viral immune system of prokaryotes to enable programmable DNA mutagenesis (Jinek et al., 2012; Cong et al., 2013; Larson et al., 2013; Mali et al., 2013). Variations of this system have since been used to perturb many other cellular process, such as transcriptional repression and enhancement (Larson et al., 2013; Kiani et al., 2014), dynamic chromatin imaging (Guan et al., 2017), single stranded RNA targeting (Abudayyeh et al., 2016), and allele-specific gene deletion (Shin et al., 2016; Monteys et al., 2017). The CRISPR/Cas system can also be used to create genetically modified animals with less difficulty and higher efficiency than traditional methods (Wang et al., 2013). This system also offers a safer alternative to RNAi-mediated gene knockdown, which requires the constant overexpression of an shRNA to mediate gene knockdown which can be toxic *in vivo* (Grimm et al., 2006; McBride et al., 2008; de Solis et al., 2015).

In order to utilize the CRISPR/Cas9 system, a gene encoding the Cas9 enzyme and a gene for the appropriate dual tracrRNA:crRNA expression cassette that serves as a single guide RNA (sgRNA) are required. The ∼20 nucleotides at the 5′ end of the sgRNA serves as the guide RNA (gRNA) that can be any sequence complementary to a genomic target location that has an adjacent protospacer adjacent motif (PAM). The PAM sequence is a short sequence adjacent to the Cas9 nuclease cut site that the Cas9 molecule requires for appropriate binding. When these two components are expressed, the sgRNA will bind to Cas9 and direct it to the sequence complementary to the gRNA, where it will then initiate a double-stranded break (DSB). To repair these breaks, cells use an error prone mechanism of non-homologous end joining (NHEJ) which can lead to disruption of function in the target gene through insertions or deletion of codons, shifts in the reading frame, or result in a premature stop codon triggering nonsense-mediated decay. Alternatively, templates of donor DNA can be provided to enable homology-directed repair (HDR) and homology-independent targeted integration (HITI) allowing for targeted insertion of genetic sequences *via* recombination (Cong et al., 2013; Mali et al., 2013; Wang et al., 2013; Suzuki et al., 2016; Zhang et al., 2017).

The CRISPR/Cas system can be delivered *in vivo via* adeno-associated virus (AAV) (Ran et al., 2015a; Swiech et al., 2015). This has the potential to be quite useful in clinical settings in which AAV is often the vector of choice for gene therapies. The ability to deliver the CRISPR/Cas system *via* AAV can also benefit the biological sciences generally. In particular, this technology can greatly benefit behavioral neuroscience by enabling selective manipulation of gene expression in specific brain regions. However, many of the exciting tools developed from CRISPR-SpCas9 technology are very bulky and do not fit within the genome packaging limits of AAV (∼4.85 kb including both ITRs). One way to overcome this technical hurdle is to take advantage of the smaller orthologs of Cas9 derived from different prokaryotic species. Three Cas9 nucleases have been discovered that have similar properties to SpCas9 but are significantly smaller. This is significant because SpCas9 is ∼4.3 kb and it barely fits into the adeno-associated viral genome when coupled with essential gene regulatory elements (i.e., promoter, 3′UTR with poly A signal sequences). However, *Streptococcus thermophilus* Cas9 (St1Cas9), *Staphylococcus aureus* Cas9 (SaCas9), and *Neisseria meningitidis* Cas9 (NmCas9) are all ∼1 kb shorter than SpCas9 (Esvelt et al., 2013; Ran et al., 2015b). Thus, with the discovery of these smaller Cas9 nucleases, AAVs can now be engineered with either of these smaller Cas9 genes and other genetic components that could include transcriptional repression domains or activator domains to create AAV–based CRISPRi and CRISPRa. They can also be rendered Cre-dependent or designed to include the gRNA expression cassettes in the same AAV vector. Another advantage of having multiple Cas9 molecules is that it enables multiplexing within a single experiment, where researchers can simultaneously target one gene for destruction and another gene for transcriptional activation. This is possible because each Cas9 ortholog utilizes distinct tracrRNAs and recognizes distinct PAM sequences (Kleinstiver et al., 2015).

For experiments that necessitate stricter temporal and/or spatial control of the system’s genome editing capabilities, it would be advantageous to have the ability to regulate expression of one or both of the system’s components. Notably, advances in science have enabled and necessitated approaches that allow for enhanced precision. This includes the ability to target very discrete cell populations for genetic manipulations within complex tissues. In particular, the CRISPR/Cas9 system has enabled the genetic modification of cells with exquisite ease and accuracy, but there are improvements that can be made with respect to its ease of deliverability and its temporal and spatial control. For example, regulating the duration the CRISPR/Cas9 system is active may reduce unwanted off-target editing that may occur at a low frequency. To that aim, we previously developed a CRISPR/Cas system with the *Streptococcus pyogenes* Cas9 enzyme (SpCas9) that enabled tightly regulated expression of the sgRNA dependent on the presence of doxycycline (Dox) *in vitro* and *in vivo* (de Solis et al., 2016). Here, we extend our previous work by generating Dox-dependent sgRNAs for SaCas9, and demonstrate strict temporal regulation both *in vitro* and *in vivo*. We previously attempted to regulate expression of SpCas9 *via* Dox from a TRE3G promoter, but found its expression to be leaky, resulting in genome editing without the presence of Dox. Here, we report the development of a Cre-loxP system to conditionally regulate the expression and genome editing of *Streptococcus aureus* Cas9 (SaCas9) *via* the expression of Cre-recombinase *in vitro* and *in vivo*. While recently published work demonstrates a similar approach using SpCas9 (Yang et al., 2017), using SaCas9 enabled us to create both single and dual AAV vectors to deliver the system. Furthermore, we demonstrate the efficacy of this system *in vivo* in CaMKIIα-Cre T29-1 mice (Tsien et al., 1996).

## Materials and Methods

### Plasmid Construction

All plasmids were constructed using standard recombinant DNA cloning techniques. pAAV-pMecp2-SpCas9-spA (pX551) was a gift from Feng Zhang [(Swiech et al., 2015); Addgene plasmid # 60957] and was utilized as the backbone for the SaCas9 vectors. P2A-HA-FLAG-HA-KASH sequence was inserted using a custom designed gBlock (Integrated DNA Technologies) between the BamHI-EcoRI sites. PX601-AAV-CMV::NLS-SaCas9-NLS-3xHA-bGHpA;U6::BsaI-sgRNA was a gift from Feng Zhang [(Ran et al., 2015a); Addgene plasmid # 61591]. SaCas9 was amplified from pX601 and cloned into the AgeI-BamHI sites of pX551, resulting in pAAV-Mecp2P-SaCas9-P2A-HAFLAGHA-KASH-pA, later referred to as Mecp2-SaCas9-HAFlagHA-KASH. An EFS promoter, amplified from our pAAV-EFS-GFP vector (Holehonnur et al., 2015), was inserted at the XbaI-AgeI sites immediately before SaCas9, within the pAAV-Mecp2P-SaCas9-P2A-HAFLAGHA-KASH-pA yielding pAAV-EFS-SaCas9-P2A-HAFLAGHA-KASH-pA, later referred to as EFS-SaCas9-HAFlagHA-KASH. An AAV-CMV-MCS3 (multi cloning site) plasmid was used as the backbone to render the SaCas9 plasmid Cre-dependent. An EFS promoter sequence was amplified using a gBlock (Integrated DNA Technologies), and this was cloned into AAV-CMV-MCS3 *via* the MluI-EcoRI sites, resulting in pAAV-EFS(no AgeI)-MCS3. Next, the NLS-SaCas9-NLS-P2A-HAFlagHA-KASH insert was cloned into the AAV-EFS(no AgeI)-MCS3 using the XbaI-AgeI sites within the MCS3, inverting the orientation of SaCas9. Two loxP sequences, the first containing loxP followed by lox2272, the second containing lox2272 followed by loxP, were amplified from custom designed gBlocks and inserted at either ends of the inverted SaCas9 sequence, using the EcoR1 and AgeI-SpeI sites, respectively. After testing our EFS-driven floxed SaCas9 [pAAV-EFS-SaCas9-HAFLAGHA-KASH-DIO(floxed)-pA] *in vitro* and seeing moderate results, we switched the EFS promoter for the stronger CMV promoter, which was cloned into the MluI-SalI sites. This required removal of the P2A-HAFlagHA-KASH to remain under the AAV genome packaging limit. Additionally, we added in a stop codon following SaCas9 with a custom oligo between the AscI-AvrII sites, resulting in pAAV-CMV-SaCas9-DIO(floxed)-pA, later referred to as CMV-SaCas9-DIO. pAAV-U6-sgRNA_(SapI)__hSyn-GFP-KASH-bGH (PX552) was a gift from the Zhang lab [(Swiech et al., 2015); Addgene plasmid # 60958] and was used as the backbone for the gRNA expression cassette. The SaCas9 compatible sgRNA expression cassette was first amplified from a gBlock and subsequently cloned in *via* the MluI-XbaI sites. The EFS promoter was PCR amplified from the pAAV-EFS-GFP (Holehonnur et al., 2015), and cloned in to the SpeI-XbaI sites after the sgRNA cassette, yielding pAAV-U6-sgRNA(SapI)_EFS-GFP-KASH-bGH, referred to here as U6-gRNA*_Empty_*-GFP-KASH. To create the Dox-inducible gRNA, a previously generated plasmid containing a Dox-inducible gRNA expression cassette for SpCas9 [AAV:ITR-U6-sgRNA_(*SapI*)_-TetR-P2A-CMV-GFP-KASH-WPRE-shortPA-ITR] (de Solis et al., 2016) was used as the backbone. The SaCas9 gRNAi expression cassettes U6/TO and H1/TO were PCR amplified from appropriately designed gBlocks (Integrated DNA Technologies) and cloned into the MluI-XbaI sites, yielding AAV:ITR-U6/TO-SaCas9sgRNA(SapI)-TetR-P2A-CMV-GFP-KASH-WPRE-shortPA-ITR and AAV:IT R-H1/TO-SaCas9sgRNA(SapI)-TetR-P2A-CMV-GFP-KASH-W PRE-shortPA-ITR, referred to as U6/TO-gRNAi*_EMPTY_*-GFP-KASH, and H1/TO-gRNAi*_EMPTY_*-GFP-KASH, here. Finally, to create our vector containing both our CMV-driven floxed SaCas9 and our constitutively expressed gRNA, we amplified the U6-driven SaCas9 gRNA expression cassette from U6-gRNA*_Empty_*-GFP-KASH and inserted it into the MluI enzyme site of pAAV-CMV-SaCas9-DIO(floxed)-pA, resulting in pAAV-U6SaCas9gRNA(SapI)-CMV-SaCas9-DIO(floxed)-pA, referred to here as U6-gRNA*_EMPTY_*-CMV-SaCas9-DIO, or U6-gRNA*_CREB_*-CMV-SaCas9-DIO, when containing our gRNA targeting the CREB locus. Sequences for our gRNAs were cloned into the SapI sites of the gRNA and gRNAi expression cassettes for each respective vector. For the first set of *in vitro* experiments the gRNAs used were as follows: EMX1-sg1: GGCCTCCCCAAAGCCTGGCCA and EMX1-sg2: TGGCCAGGCTTTGGGGAGGCC; for the second set of *in vitro* experiments and all *in vivo* studies, the gRNA sequence used was CREB: GGAGCAGACAACCAGCAGAG.

### Immunocytochemistry

Glass coverslips were placed in 24-well cell culture plates and coated with poly-l-lysine overnight (0.1 mg/ml; Sigma). The next day, coverslips were rinsed thrice with 1× PBS (pH 7.4) and seeded with 293FT cells to 60% confluency. Twenty-four hours later, cells were transfected with plasmids designed to express SaCas9, using Lipofectamine 2000 (Invitrogen), according to the manufacturer’s instructions. Six hours post transfection, the existing media was replaced with fresh media and the ICC was performed 24 hours post transfection. The primary antibodies used were anti-Cas9 (1:500; EnCor Biotechnology, #MCA-6F7); anti-Flag (1:200; Sigma); anti-HA (1:1000; Covance MMS:101P), and the secondary antibody was TxRed secondary antibody (1:000; Life Technologies). DAPI (1:25,000; Thermo Fisher) was applied last. The ICC images were taken using a fluorescence microscope at 400× magnification (Olympus, BX51).

### Assessment of *in vitro* Genome Editing

293FT cells (Invitrogen) or Neuro-2a cells (N2A; ATCC) were grown at a confluency of 60–65% in a 24-well plate. Cells were transfected with a plasmid containing a Cas9 transgene and a plasmid containing a gRNA expression cassette in a 1:1 ratio using the Lipofectamine 2000 (Invitrogen) protocol. The gRNA expression cassette either was designed to target the human EMX1 locus (EMX1-sg1/EMX1-sg2) or the mouse CREB locus or was a gRNA expression cassette without a gRNA sequence (Empty). Editing was assessed in these cell lines due to their ability to be transfected efficiently thus facilitating the assessment of genome editing on a population of cells. For the Dox-inducible experiments, the media was replaced 6 hours post transfection and 10 μg/mL Dox (Clontech) was added to some samples. To assess editing efficiency of the Cre-dependent plasmids, the Cas9, gRNA, and pBS185 CMV-Cre plasmids were transfected in a 1:1:1 ratio into 293FT cells. Cells were harvested 96 h post transfection and collected by centrifugation (10 min; 14,000 RPM). Genomic DNA was extracted using the Genomic DNA Extraction Kit (Epoch Biolabs GenCatch^TM^; Catalog # 24-60050). The region containing the gRNA target site was PCR amplified from the genomic DNA using appropriately designed primers (EMX1-sg1 FP: CCCATAGGGAAGGGGGACAC, EMX1-sg1 RP: GGGCTCCCATCACATCAACC, EMX1-sg2 FP: GGGCTCCCATCACATCAACC EMX1-sg2 RP: CCCATAGGGAAGGGGGACAC, CREB FP: GAGCAAGTTGAGTAAAAGATCAGCTG, CREB RP: CAGTTGTCATCATTGCCTTGAGAGGGCCAATTC). The resultant PCR amplified DNA was screened using the resolvase-based mutation detection kit (Clontech, Cat #631443). Briefly, DNA was amplified using the following cycling conditions [98°C, 2 min; (98°C, 10 s; 60°C, 15 s; 68°C, 1 min) × 35 cycles, 72°C, 45 s]. After the amplification was confirmed and quantified using gel electrophoresis (1.5% agarose, 1× TAE gel), DNA hybridization was performed on 200 ng of DNA at the following parameters [95°C, 5 min; 95–85°C, 2°C/s^∗^; 85–25°C, 0.1°C/s^∗^; cool to 4°C (^∗^rate of temperature decrease)], followed by incubation with resolvase for 15 min at 37°C. The digested product was electrophoresed on a 2.0% agarose, 0.5 × TBE gel to determine if genome editing occurred. The amount of editing was quantified using ImageJ (U.S. National Institutes of Health, Bethesda, MD, United States^1^). Editing efficiency refers to the average amount of edited DNA compared to the unedited DNA per condition. No normalization was applied. All untransfected controls (UTC) or empty conditions exhibited no editing. The editing displayed is the average of samples within a particular condition.

### Viral Production, Purification, and Titering

Procedures were carried out as previously described (Holehonnur et al., 2015). AAV2 genome plasmids were pseudotyped as DJ serotype using a triple-transfection, helper-free method. AAV genome, DJ RC serotype, and pHelper plasmids were transfected into 293FT cells using TurboFect Transfection Reagent (Thermo Scientific; R0532) according to the manufacturer’s instructions for AAV production. Purified AAV was titered using a quantitative-PCR based titering method as previously described (Holehonnur et al., 2015). All viruses were titered using custom DNA primers for either SaCas9 or GFP. The Cas9 primers were used to titer viruses made from the following vectors: pAAV-Mecp2-NLS-SaCas9-NLS-P2A-HAFlagHA-KASH-pA, pAAV-EFS-NLS-SaCas9-NLS-P2A-HAFlagHA-KASH-pA, pAAV-CMV-NLS-SaCas9-NLS-DIO-pA, pAAV-U6-sgRNA*_CREB/EMPTY_*_CMV-NLS-SaCas9-NLS-DIO-pA (Cas9 primers: SaCas9 QR FP; CAACGCCGACCTGTACAACG, SaCas9 QR RP; TCTGCTTCAGGGTGGGCTTC). GFP primers were used to titer viruses made from the following vectors: pAAV-U6-sgRNA*_CREB/EMPTY_*_EFS-GFP-KASH-pA and pAAV-H1/TO-sgRNA*_CREB/EMPTY_*_CMV-TetR-P2A-GFP-KASH-pA (GFP primers: GFP FP qRT; AAGCTGACCCTGAAGTTCATCTGC, GFP RP qRT; CTTGTAGTTGCCGTCGTCCTTGAA).

### Viral Infusion

Viral infusions targeting the mouse basal and lateral amygdala complex (BLA) were performed as described previously (de Solis et al., 2016). Mice were anesthetized with an intraperitoneal injection of ketamine (100 mg/kg) and xylazine (10 mg/kg) prior to stereotaxic surgery. For the constitutively expressed system, AAV2/DJ-P_*mecp*2_-SaCas9-P2A-HAFlagHA-KASH and AAV2/DJ-U6-SaCas9gRNA*_CREB/EMPTY_*_EFS-GFP-KASH-pA were co-infused bilaterally at a titer of 5.0E12 GC/mL (1 μL/side). For Dox-induced experiments, AAV2/DJ-P_*mecp*2_-SaCas9-P2A-HAFlagHA-KASH-pA and AAV2/DJ-gRNAi*_CREB/EMPTY_*_CMV-GFP-KASH-pA, or AAV2/DJ-U6-SaCas9gRNA*_CREB/EMPTY_*_EFS-GFP-KASH-pA, were co-infused bilaterally at a titer of 5.0E12 GC/mL (1 μL/side). Mice were supplied Dox through their food (Dox 1 g/kg; Bio-Serv). For the Cre-dependent system, AAV2/DJ-P*_CMV_*-SaCas9-DIO and AAV2/DJ-gRNA*_CREB/EMPTY_*_CMV-GFP-KASH-pA, or AAV2/DJ-U6-SaCas9gRNA*_CREB/EMPTY_*-CMV-SaCas9-DIO were co-infused bilaterally into the BLA of CaMKIIα-Cre transgenic mice or wildtype C57BL/6 mice at a titer of 3.5E12 GC/mL (1 μL/ side), 5.0E12 GC/mL (1 μL/ side), and 8.0E12 GC/mL (1 μL/ side), respectively.

### Assessment of *in vivo* Genome Editing

Mice were lightly anesthetized with CO_2_, the brain was quickly removed, frozen with powdered dry ice and stored at -80°C until further processing. To obtain coronal sections that contained the amygdala, tissue was mounted and sliced using a cryostat (Thermo Scientific). Multiple 200 μm coronal slices were taken that contained the amygdala from each animal. Using a 0.5 mm punch tool, the amygdala transduced tissue was dissected from each slice that contained viral-mediated GFP signal. The punched tissue was stored at -80°C until DNA purification. Genomic DNA was purified in the same manner as the *in vitro* experiments and subsequently amplified using the Clontech 639123 Advantage^®^ HF 2 PCR kit [94°C, 1 min (94°C, 30 s, 64°C, 30 s, 68°C, 1 min) × 35 cycles, 68°C, 3 min]. Amplification was confirmed and quantified using gel electrophoresis (1.5% agarose, 1× TAE gel). To assess genome editing, the samples were digested with the restriction enzyme BstXI. The digested product was then electrophoresed on a 1.5% agarose TAE gel. Editing was quantified using ImageJ. Editing efficiency refers to the average amount of edited DNA compared to the unedited DNA per condition. No normalization was applied. The editing displayed is the average of samples within a particular condition.

### Subjects

Adult C57BL/6 mice and adult CaMKIIα-Cre transgenic mice [(Tsien et al., 1996); The Jackson Laboratory Stock No: 005359| T29-1], were housed individually and maintained on a 12 h light/dark cycle. Food and water were provided *ad libitum* throughout the experiments. Animal use procedures were in accordance with the National Institutes of Health Guide for the Care and Use of Laboratory Animals and were approved by the University of Texas at Dallas Animal Care and Use Committee.

### Statistical Analysis

Quantified genome editing data was analyzed using a non-parametric Kruskal–Wallis individual sample comparison or multiple sample comparison. A two-tailed *t*-test assuming unequal variances was used to compare vectors differing in promoters, sgRNAs, or expression conditions (i.e., the Dox-inducible and Cre-dependent systems). A probability threshold of 0.05 was used for all statistical analyses.

## Results

### SaCas9/CRISPR System Exhibits Efficient Genome Editing *in vitro*

**FIGURE 1:**
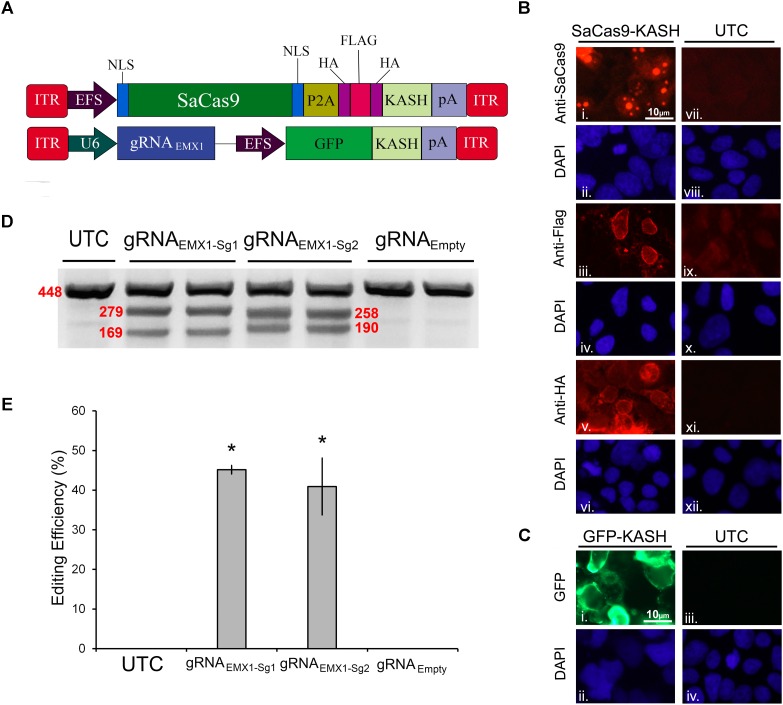
SaCas9/CRISPR systemexhibits efficient genome editing *in vitro*. **(A)** AAV vector maps depicting EFS-SaCas9-HAFlagHA-KASH and gRNA_*EMX*1-*sg*1_-GFP-KASH. EFS-SaCas9-HAFlagHA-KASH is an AAV vector that consists of an EFS promoter controlling the expression of the coding regions of SaCas9 followed by a self-cleaving P2A peptide sequence, and then an HA Flag HA epitope tag fused to a KASH domain. gRNA_*EMX*1-*sg*1_-GFP-KASH is a dual transgene containing AAV vector with a U6-driven SaCas9 sgRNA expression cassette and a GFP-KASH transgene under the control of an EFS promoter. **(B)** EFS-SaCas9-HAFlagHA-KASH (SaCas9-KASH) viral plasmid was transfected into 293FT cells. For a control, similar cells were not transfected [untransfected control (UTC)]. Forty-eight hours later, an ICC for SaCas9, HA, and Flag expression was conducted. Images depict cells examined for SaCas9 (i and vii), Flag (iii and ix), and HA (v and xi) immunostaining. Similar fields of view also depict DAPI-stained nuclei [SaCas9 (ii and viii), Flag (iii and ix), and HA (vi and xii)]. Expression patterns followed expectation for cells transfected with SaCas9-KASH, with Flag (iii) and HA (v) expression appearing perinuclear and SaCas9 expression predominately in the nucleus (i). Notably UTCs do not exhibit signal for SaCas9 (vii), Flag (ix), and HA (xi) as expected. **(C)** gRNA*_Empty_*-GFP-KASH (GFP-KASH) viral plasmid was transfected into 293FT cells. For a control, similar cells were not transfected [untransfected control (UTC)]. Forty-eight hours later, native GFP fluorescence was observed for GFP-KASH expression. Similar fields of view also depict DAPI-stained nuclei [GFP (ii and iv). Expression patterns followed expectation for cells transfected with GFP-KASH, with GFP expression appearing perinuclear (i). Notably UTCs do not exhibit GFP expression (iii). **(D)** 293FT cells were co-transfected with EFS-SaCas9-HAFlagHA-KASH and U6-gRNA_*EMX*1-*sg*1_-GFP-KASH/U6-gRNA_*EMX*1-*sg*2_-GFP-KASH/U6-gRNA*_Empty_*-GFP-KASH. Ninety-six hours post transfection, cells were harvested and genomic DNA was isolated and purified. A 448 bp region of the EMX1 locus including the site targeted for editing *via* the two gRNA_EMX1_ was PCR amplified from the genomic DNA and analyzed for genome editing with a resolvase-based mutation detection kit (Clontech). Processed samples were electrophoresed on a 2% agarose, 0.5 × TBE gel stained with ethidium bromide. The top band on the gel is uncut DNA at 448 bp in length while the two smaller bands are edited DNA at 279 bp and 169 bp in length for EMX1-sg1, 258 and 190 bp in length for EMX1-sg2. Editing occurred in lanes 2–5 which received both EFS-SaCas9-HAFlagHA-KASH and either U6-gRNA_*EMX*1-*sg*1_-GFP-KASH or U6-gRNA_*EMX*1-*sg*2_-GFP-KASH, but not in lanes 6–7 as expected, which received EFS-SaCas9-HAFlagHA-KASH and U6-gRNA*_Empty_*-GFP-KASH. Similar results were obtained in four independent samples per group.**(E)** Optic densities of the bands were quantified using ImageJ. Editing efficiencies of gRNA_*EMX*1-*sg*1_ and gRNA_*EMX*1-*sg*2_ were 45 and 41%, respectively [*H*(2, *N* = 12) = 7.652174, *p* = 0.0218, Kruskal–Wallis]. There was no significant difference between the two gRNAs in their editing efficiency [*t*(6) = 0.5860, *p* = 0.5991]. ^∗^*p* < 0.05, compared to untransfected control (UTC) and Empty controls which were both zero, error bars = standard error of the mean.

In our first set of experiments, we focused on designing a CRISPR/SaCas9 system suitable for AAV delivery. We aimed to construct a constitutively expressing SaCas9 system split between two viral plasmids. The first AAV plasmid contains an EF-1α short (EFS) promoter controlling expression of the SaCas9 coding region fused to a self-cleaving P2A sequence attached to an HA-Flag-HA epitope tagged Klarsicht, ANC-1, Syne homology (KASH) domain. The P2A sequence enables the SaCas9 and the epitope tagged KASH domain to be formed as two independent proteins from the same cistron. Tagged KASH domains have been shown to localize to the nuclear membrane enabling nuclei from transduced tissue to be collected (Swiech et al., 2015). Transduced cells can be separated from untransduced cells *via* fluorescent-activated cell sorting (FACS) analysis. This is especially useful when these viruses are used to transduce intact brain tissue because isolating intact neuronal cell bodies can be difficult from the adult brain. The second AAV plasmid contains a constitutively expressing gRNA expression cassette controlled from a U6 promoter. It also contains a transgene designed to express green fluorescent protein (GFP), fused to a KASH domain from an EFS promoter (Figure 1A). An immunocytochemistry (ICC) experiment was performed on 293FT cells that were transfected with the EFS-SaCas9-HAFlagHA-KASH plasmid to confirm expression of the transgene (Figure 1B). We were able to detect the expression of the HA and Flag epitopes, and the SaCas9 with the appropriate antibodies. The localization patterns for the KASH fused HA and Flag epitopes, display a pattern of expression that is consistent with staining that appears localized to the outer nuclear membrane and endoplasmic reticulum. SaCas9 appeared to localize predominately inside the nucleus to what appear to be nucleoli. Similarly, a transfection of the U6-gRNA*_Empty_*-GFP-KASH plasmid was performed to confirm that expression of GFP was perinuclear (Figure 1C).

To examine the genome editing capabilities of our system, 293FT cells were co-transfected with the SaCas9 and gRNA viral plasmids. The gRNA plasmids either encoded gRNAs designed to target the human empty spiracles homeobox 1 (EMX) locus (EMX1-sg1 or EMX1-sg2) or did not contain a gRNA (Empty) as a control. Ninety-six hours post transfection, the cells were harvested and collected by centrifugation. Genomic DNA was isolated and a 448-bp region from the EMX1 locus was PCR amplified and examined for Cas9-mediated editing utilizing a resolvase-based screening method. Using this assay, if no editing occurs, a single band will be visible on the gel. When editing does occur, the DNA is cut at the location of the editing, resulting in two smaller bands 279 and 169 bp in length for EMX1-sg1, 258 and 190 bp in length for EMX1-sg2 (Figure 1D). Cells that received the EFS-SaCas9-HAFlagHA-KASH and U6-gRNA*_Empty_*-GFP-KASH vectors did not exhibit editing of the EMX1 locus. However, cells transfected with EFS-SaCas9-HAFlagHA-KASH and U6-gRNA_*EMX*1-*sg*1_-GFP-KASH or U6-gRNA_*EMX*1-*sg*2_-GFP-KASH gRNA displayed an average of 45 and 41% editing, respectively [*H*(2, *N* = 12) = 7.652174, *p* = 0.0218, Kruskal–Wallis]. Differences in editing efficiency between the two gRNAs were not significant [*t*(6) = 0.5860, *p* = 0.5991] (Figure 1E). Genome editing percentages were averaged from four independent samples per group (*n* = 4).

### SaCas9-Compatible Inducible gRNA Vectors Exhibit Doxycycline-Inducible Genome Editing *in vitro*

**FIGURE 2:**
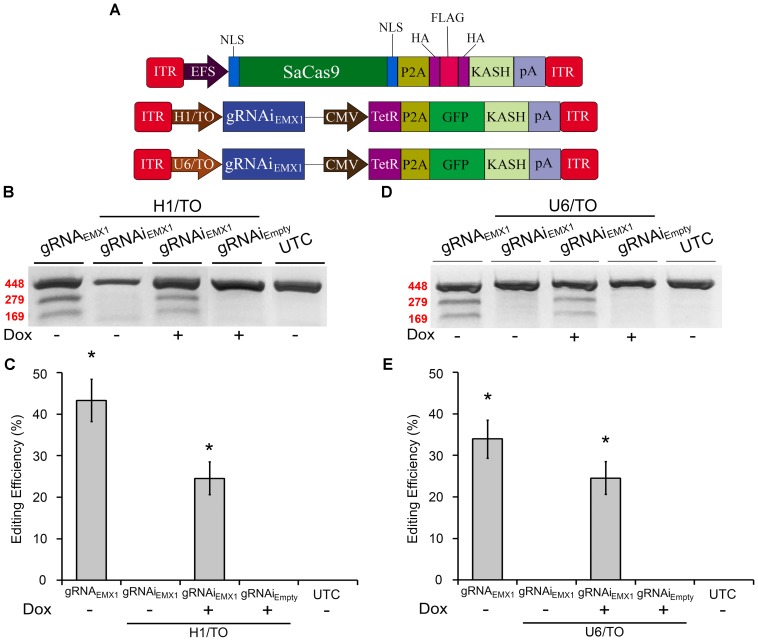
SaCas9-compatible inducible gRNA vectors exhibits doxycycline-inducible genome editing *in vitro*. **(A)** AAV vector maps depicting EFS-SaCas9-HAFlagHA-KASH, H1/TO-gRNAi_*EMX*1-*sg*1_-GFP-KASH, and U6/TO-gRNAi_*EMX*1-*sg*1_-GFP-KASH. **(B)** Gel images to detect genome editing. Cells were co-transfected with EFS-SaCas9-HAFlagHA-KASH and U6-gRNA_*EMX*1-*sg*1_-GFP-KASH or H1/TO-gRNAi_*EMX*1-*sg*1_-GFP-KASH or H1/TO-gRNAi**_EMPTY_** -GFP-KASH in the presence or absence of Dox. Samples were processed as described above to detect genome editing. “ + ” or “–” indicates the absence or presence of Dox in the cell media during the 96-hour incubation period. Similar results were obtained in four independent samples per group. **(C)** Quantification of samples transfected with our inducible system driven by the H1/TO promoter yielded a 24.5% editing efficiency, and editing was dependent on the presence of Dox [*H*(2, *N* = 12) = 10.202, *p* = 0.0061, Kruskal–Wallis]. Samples receiving the constitutively expressed system (EFS-SaCas9-HAFlagHA-KASH, +U6-gRNA_*EMX*1-*sg*1_) exhibited 33.8% genome editing. **(D)** Gel images to detect genome editing. Cells were co-transfected with EFS-SaCas9-HAFlagHA-KASH and U6-gRNA_*EMX*1-*sg*1_-GFP-KASH or U6/TO-gRNAi_*EMX*1-*sg*1_-GFP-KASH or U6/TO-gRNAi**_EMPTY_**-GFP-KASH were processed as described above. “ + ” or “–” indicates the absence or presence of Dox in the cell media during the 96-h incubation period. **(E)** Samples transfected with our inducible system controlled by the U6/TO promoter yielded around 24.5% editing efficiency, and editing was dependent on the presence of Dox [*H*(2, *N* = 12) = 8.289, *p* = 0.0158, Kruskal–Wallis]. ^∗^*p* < 0.05 compared to untransfected control (UTC) and Empty controls which were both zero, error bars = standard error of the mean.

We previously created an SpCas9-compatible Dox-inducible gRNA AAV vector that enabled efficient Dox-dependent genome editing (de Solis et al., 2016). Here, we generated two SaCas9-compatible inducible gRNA (gRNAi) AAV vectors and examined their ability to regulate genome editing in a Dox-inducible manner. These vectors possessed gRNA expression cassettes controlled by either a hybrid H1 Tet operator (H1/TO) promoter or a hybrid U6 Tet operator (U6/TO) promoter. These vectors also contain a constitutively expressed Tet Repressor (TetR) fused to a self-cleaving P2A sequence, in frame with a KASH-tagged GFP, controlled by a CMV promoter. In the absence of Dox, TetR binds to the Tet operator and represses gRNA transcription. When Dox is present it will bind the Tet repressor, preventing it from binding the Tet Operator, thus allowing gRNA expression to commence (Figure 2A). To test these systems *in vitro*, 293FT cells were transfected with the appropriate SaCas9 and gRNAi plasmids. Ninety-six hours post-transfection, the genomic DNA was isolated and genome editing efficiency was assessed as described above (Figures 2B,D). Cells receiving our constitutively active editing system exhibited robust editing, as expected. Cells that received H1/TO or U6/TO gRNAi vectors exhibited ∼25% editing when Dox was present [H1/TO, *H*(2, *N* = 12) = 10.202, *p* = 0.0061, Kruskal–Wallis; U6/TO, *H*(2, *N* = 12) = 8.289 *p* = 0.0158, Kruskal–Wallis]. No editing was observed in the absence of Dox for our inducible systems, or in samples transfected with the Empty control gRNAi vector (Figures 2C,E). Genome editing percentages were averaged from four independent samples per group (*n* = 4).

### A Double-Inverted Orientation (DIO) SaCas9 AAV Vector Allows for Cre-Dependent Genome Editing *in vitro*

**FIGURE 3:**
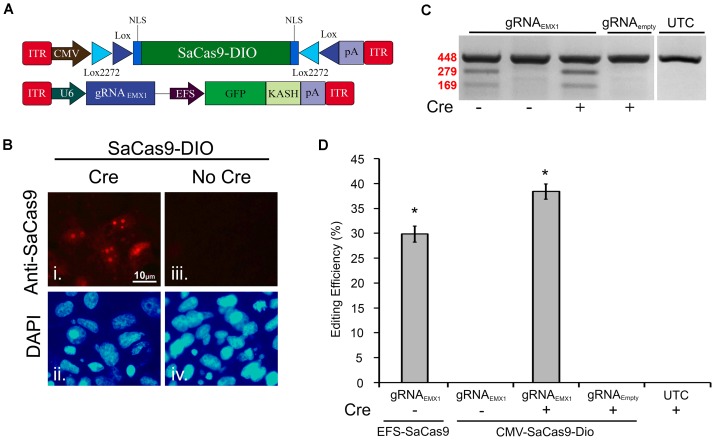
A double-inverted orientation (DIO) SaCas9 AAV vector allows for Cre-dependent genome editing *in vitro*. **(A)** AAV vector maps depicting our floxed-SaCas9 (CMV-SaCas9-DIO) and gRNA_*EMX*1-*sg*1_-GFP-KASH. **(B)** SaCas9-DOP viral plasmid was transfected into 293FT cells with or without a plasmid that is designed to express Cre recombinase. Forty-eight hours later an ICC for SaCas9 expression was conducted. Images depict cells examined for SaCas9 expression. Similar fields of view also depict DAPI stained nuclei (ii and iv). Expression patterns followed expectation for cells transfected with SaCas9-DIO and Cre, with SaCas9 expression appearing nuclear (i). Notably cells that received SaCas9-DIO but did not receive the Cre plasmid do not exhibit SaCas9 expression (iii). **(C)** Gel images to detect genome editing. Presence or absence of Cre is indicated “+” or “-” underneath the gel lane. Samples depicted in Lane 1 received our constitutively expressed system as the positive control. When transfected with gRNA_*EMX*1-*sg*1_-GFP-KASH (lanes 1–3) and the floxed-SaCas9 (CMV-SaCas9-DIO) (lanes 2–4), editing is only seen in the presence of Cre and the appropriate gRNA (lane 3). Similar results were obtained in four independent samples per group. **(D)** Quantification of the constitutively expressed system exhibited an average editing of 29.8%, while the Cre-dependent system exhibited an average editing of 38.39% [*H*( 2, *N* = 12) = 10.202, *p* = 0.0061, Kruskal–Wallis], in the presence of Cre.^∗^*p* < 0.05, compared to untransfected control (UTC) and Empty controls which were both zero, error bars = standard error of the mean.

The Cre-Lox system has been widely used to make precise Cre-dependent genetic modifications. Transgenes flanked with LoxP, and Lox2272, sites on either side will be inverted when exposed to Cre-recombinase. This can be used to activate desired transgenes in a Cre-dependent manner. Furthermore, if Cre expression is limited to specific cell types of an organism, or supplied at a specific time, the activation of the transgene will be limited to this specific region. Regulating genome editing capabilities in this way could provide researchers with much greater spatial and temporal editing precision. Recent research has shown the potential of the Cre-Lox system in regulating SpCas9’s and SaCas9’s genome editing capabilities *via* floxed-gRNA vectors (Yang et al., 2017). Here, we extend this work by generating a floxed-SaCas9 within an AAV genome. Initially, we built our system by adding the necessary lox sites to our EFS-SaCas9-P2A-HAFlagHA-KASH vector; however, this vector did not efficiently edit DNA *in vitro* (data not shown). We then switched out our EFS promoter for a CMV promoter and removed the P2A-HAFlagHA-KASH domains in order to keep the AAV genome within the optimal packaging limit (Figure 3A). First, CMV-SaCas9-DIO was transfected into 293FT cells with or without a plasmid designed to express Cre-recombinase. Forty-eight hours post transfection, an ICC experiment was performed to detect SaCas9 expression with anti-SaCas9 antibodies. Expression of SaCas9 only occurred when Cre was present, indicating the SaCas9-DIO was functioning correctly (Figure 3B).

EFS-SaCas9-P2A-HAFlagHA-KASH with U6-gRNA_*EMX*1-*sg*1_-GFP-KASH and CMV-SaCas9-DIO along with U6-gRNA_*EMX*1-*sg*1_ or U6-gRNA*_Empty_*-GFP-KASH were transfected into 293FT cells with or without a plasmid designed to express Cre-recombinase. Ninety-six hours post transfection, the cells were harvested and genomic DNA was purified and isolated as previously described. As expected, editing was only seen in the constitutively expressed system and in the Cre-dependent system when Cre was present (Figure 3C). Similar results were obtained in four independent samples per group (*n* = 4). Cells transfected with our floxed-SaCas9 (CMV-SaCas9-Dio) and gRNA_*EMX*1-*sg*1_-GFP-KASH displayed editing only in the presence of Cre (∼39%) [*H*(2, *N* = 12) = 10.202, ^∗^*p* = 0.0061, Kruskal–Wallis], which was significantly higher than our positive control (29.8%), *t*(6) = -3.8105, *p* = 0.0089 (Figure 3D). The greater genome editing seen in our Cre-dependent system may be due to the stronger CMV promoter in comparison to EFS promoter in the constitutively active system.

### The AAV-SaCas9-Mediated System Efficiently Targets the CREB Locus *in vitro*

**FIGURE 4:**
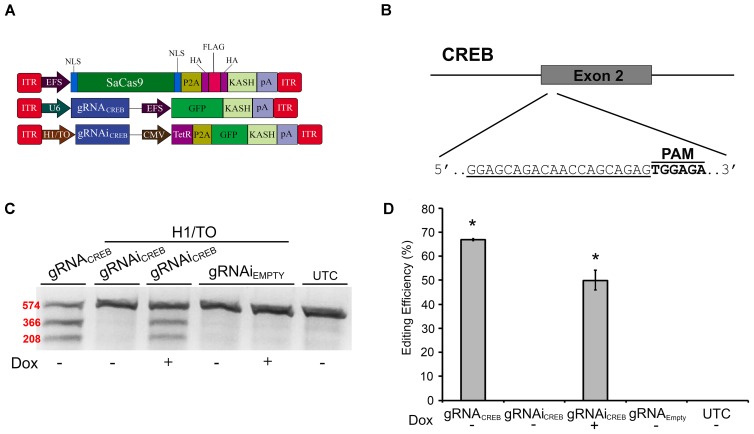
SaCas9 doxycycline system efficiently targets the CREB locus *in vitro*. **(A)** AAV vector maps depicting EFS-SaCas9-HAFlagHA-KASH, U6-gRNA*_CREB_*-GFP-KASH, and H1/TO-gRNAi*_CREB_*-GFP-KASH. **(B)** The approximate location of the CREB gRNA-binding site in the CREB locus. The gRNA, or target sequence, is underlined with the PAM sequence specified. **(C)** Cells were transfected with the gRNA vectors as denoted along with EFS-SaCas9-HAFlagHA-KASH. Ninety-six hours post transfection, samples were processed as previously described and evaluated for genome editing. The top band is 574 bp, while the two lower bands are of 366 and 208 bp in length, respectively. Our constitutively expressed gRNA was used as the positive control. Cells transfected with H1/TO-gRNAi*_CREB_*-GFP-KASH exhibited editing only in the presence of Dox. No editing was seen in the absence of Dox, or when cells were transfected with H1/TO-gRNAi**_EMPTY_**-GFP-KASH. Similar results were obtained in three independent samples per group. **(D)** Quantification showed that the constitutively expressed system had an editing efficiency of 66.8% and, in the presence of Dox, the inducible system had an average editing of 49.8% editing [*H*(3, *N* = 13) = 11.40000, *p* = 0.0097, Kruskal–Wallis].^∗^*p* < 0.05, compared to untransfected control (UTC) and Empty controls which were both zero, error bars = standard error of the mean.

Prior to testing our systems *in vivo* in mice, we needed to switch gRNAs because our EMX1 gRNAs were specific to the human genome. We designed a gRNA to target the second exon of the mouse CREB gene (Figures 4A,B) and validated its efficacy in mouse Neuro-2a (N2A) cells. We chose to target the CREB locus because it is a well-studied gene, known for its important role in neural plasticity (Martin et al., 1997; Silva et al., 1998). First, we tested this gRNA in our non-inducible and Dox-inducible system. For this, N2A cells were transfected with EFS-SaCas9-HAFlagHA-KASH and gRNAi*_CREB_*-GFP-KASH or gRNAi**_EMPTY_**-GFP-KASH in the presence or absence of Dox. Cells were harvested 96 hours later and processed as described above. Cells that were transfected with gRNAi*_CREB_*-GFP-KASH exhibited an average of 49.8% editing in the presence of Dox, and no editing in the absence of Dox [*H*(3, *N* = 13) = 11.400, *p* = 0.0097, Kruskall–Wallis] (Figures 4C,D). The constitutively expressed gRNA*_CREB_*-GFP-KASH with EFS-SaCas9-HAFlagHA-KASH exhibited an average of 66.8% editing [*t*(2) = 4.835, *p* = 0.0040]. Genome editing percentages were averaged from three independent samples per group.

**FIGURE 5:**
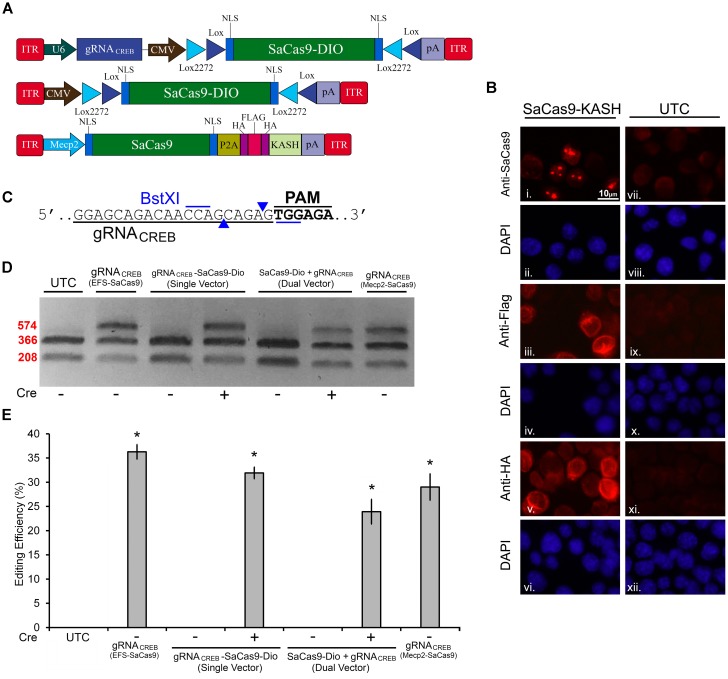
SaCas9 Cre-dependent system efficiently targets the CREB locus *in vitro*. **(A)** AAV vector maps for Cre-dependent single and dual vector systems and Mecp2-SaCas9-HAFlagHA-KASH **(B)** Mecp2-SaCas9-HAFlagHA-KASH (SaCas9-KASH) viral plasmid was transfected into N2A cells. For a control, similar cells were not transfected [untransfected control (UTC)]. Forty-eight hours later, an ICC for SaCas9, HA, and Flag expression was conducted. Images depict cells examined for SaCas9 (i and vii), Flag (iii and ix), and HA (v and xi) immunostaining. Similar fields of view also depict DAPI stained nuclei (SaCas9 (ii and viii), Flag (iii and ix), and HA (vi and xii). Expression patterns followed expectation for cells transfected with SaCas9-KASH, with Flag (iii) and HA (v) expression appearing perinuclear and SaCas9 expression predominately in the nucleus (i). Notably, UTCs do not exhibit signal for SaCas9 (vii), Flag (ix) and HA (xi) as expected. **(C)** BstXI restriction enzyme was used for the detection of genome editing from *in vivo* samples. The gRNA is shown along with the binding sites of the BstXI enzyme which overlaps with the Cas9 cutting site, and therefore, the enzyme site will likely be destroyed if editing occurs at this locus. **(D)** Quantification of genome editing for the dual and single vector Cre-dependent systems, as well as Mecp2-SaCas9-HAFlagHA-KASH. Samples transfected with our constitutively expressed system yielded 36.3% editing. Samples transfected with the dual vector Cre-dependent system, CMV-SaCas9-DIO and gRNA*_CREB_*-GFP-KASH, exhibited an editing efficiency of 23.9%. The single vector Cre-dependent system, gRNA*_CREB_*-CMV-SaCas9-DIO exhibited an editing efficiency of 31.9%, which was significantly more than the dual vector Cre-dependent system (23.9%) [*t*(5) = 4.1385, *p* = 0.0090]. Differences between the EFS-driven and Mecp2-driven constitutively expressed systems did not reach significance [*t*(4) = 0.9713, *p* = 0.386]. Genome editing percentages were averaged from four independent samples per group in **(E)**. ^∗^*p* < 0.05, compared to untransfected control (UTC) which were zero, error bars = standard error of the mean.

Next, we built a single AAV vector harboring both a Cre-dependent SaCas9 transgene and a constitutively expressed gRNA expression cassette. We tested this vector alongside our dual vector system for Cre-dependent editing of the CREB locus in mouse N2A cells. Additionally, we tested a vector using the Mecp2 promoter to drive our constitutively expressed SaCas9 (Figure 5A). This Mecp2 promoter has been previously shown to direct neuron specific expression when used within AAV (Swiech et al., 2015). An ICC was performed to confirm expression of the SaCas9-HAFlag-HA-KASH transgene from the MecP2 promoter using antibodies for HA, Flag, and SaCas9. Expression patterns again confirmed our expectations that the HA and Flag epitopes appear to localize to the outer nuclear and endoplasmic reticulum membranes and SaCas9 localization predominantly occurring in the nucleus (Figure 5B). DNA was then isolated and examined for editing *via* PCR/restriction fragment length polymorphism (RFLP), taking advantage of a BstXI restriction enzyme site that overlaps with the SaCas9 cut site (Figure 5C). In the absence of editing, BstXI will cut 100% of the CREB amplicon. In the instance of editing, the BstXI site is destroyed and BstXI would lose the ability to cut at that locus, leading to an undigested band on the gel (Figure 5D), which contains edited DNA. We utilized this method to detect genome editing of the CREB locus from our *in vivo* samples because we found the resolvase method produced too much background on these samples (data not shown). Notably, we utilized a similar strategy previously to observe editing at the Tet2 locus (de Solis et al., 2016). N2A cells were transfected with EFS-SaCas9-HAFlagHA-KASH and gRNA*_CREB_*-GFP-KASH, CMV-SaCas9-DIO and gRNA*_CREB_*-GFP-KASH, gRNA*_CREB_*-CMV-SaCas9-DIO, or Mecp2-SaCas9-HAFlagHA-KASH and gRNA*_CREB_*-GFP-KASH either in the presence or absence of Cre. When examined for editing 96 h post transfection, we observed that cells transfected with the EFS-driven constitutively expressed system exhibited 36.3% editing, the dual vector Cre-dependent system exhibited 23.9% editing, the single vector Cre-dependent system exhibited 31.9% editing, and finally the Mecp2-driven constitutively expressed system exhibited 29% editing [*H*(3, *N* = 16) = 12.985, *p* = 0.0047, Kruskal–Wallis] (Figure 5E). The dual vector Cre-dependent system exhibited significantly less editing than the EFS constitutively expressed system [*t*(5) = 4.1385, *p* = 0.0090]. The levels of editing between the single vector Cre-dependent system and the constitutively expressed system did not reach significance but there was a trend (31.9%) [*t*(6) = 2.233, *p* = 0.0670]. Differences in editing between the EFS-driven and Mecp2-driven constitutively expressed systems did not reach significance [*t*(4) = 0.971, *p* = 0.3865]. Genome editing percentages were averaged from four independent samples per group.

### AAV-Mediated Constitutively Expressed and Dox-Inducible SaCas9/CRISPR Systems Exhibit Robust Editing *in vivo*

**FIGURE 6:**
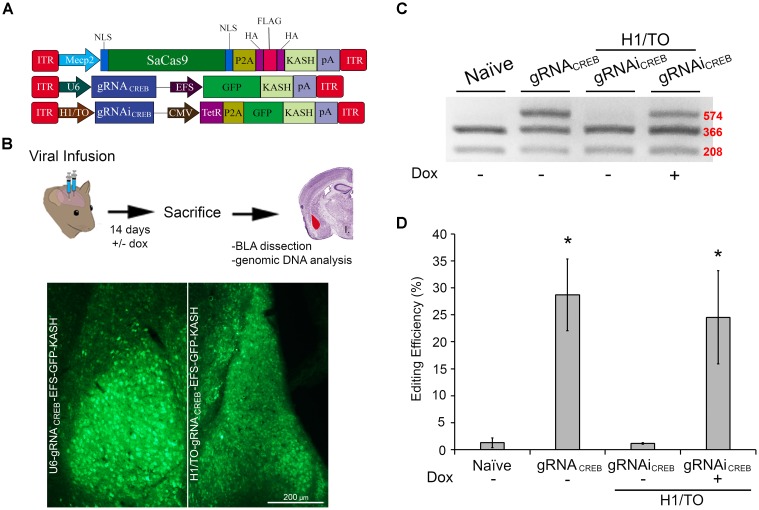
AAV-mediated constitutively expressed and Dox-inducible SaCas9/CRISPR systems exhibit robust editing *in vivo*. **(A)** AAV vector maps depicting Mecp2-SaCas9-HAFlagHA-KASH, U6-gRNA*_CREB_*-GFP-KASH and H1/TO- gRNA*_CREB_*-GFP-KASH. **(B)** Timeline from viral infusion into BLA of mice to BLA dissection, genomic DNA isolation, and RFLP analysis and images of GFP expression of transduced BLA tissue. Mice were bilaterally infused with AAV/DJ-P_*mecp*2_-SaCas9 and AAV/DJ-gRNA*_CREB_*, or AAV/DJ-P_*mecp*2_-SaCas9 and AAV/DJ-gRNAi*_CREB_*. Images depict robust viral transduction within the BLA. **(C)** Gel showing the genome editing efficiencies in the presence or absence of Dox. With our BstXI restriction enzyme digest, edited DNA is represented by the top band because editing by SaCas9 modifies BstXI’s binding site, resulting in an incomplete digest. Similar results were obtained in six independent samples per group. **(D)** Quantification showing editing percentages. When samples are transduced with AAV/DJ-P_*mecp*2_-SaCas9 and AAV/DJ-gRNA*_CREB_* editing efficiency was 34.4% [*t*(5) = 4.226, *p* = 0.0083]. When transduced with AAV/DJ-P_*mecp*2_-SaCas9 and AAV/DJ-gRNAi*_CREB_*, editing is seen only in the presence of Dox (25.2%) [*t*(5) = 3.347, *p* = 0.0204]. There was no significant difference between the constitutively expressed and Dox-inducible systems [*t*(10) = 0.477, *p* = 0.6434]. ^∗^*p* < 0.05, compared to naive, error bars = standard error of the mean.

We wanted to assess if our SaCas9 gRNAi system could be used *in vivo*, in a Dox-inducible manner. In these experiments, we used our SaCas9 vector where SaCas9 was controlled by the neuronal specific promoter Mecp2 (Figure 6A). We produced AAV/DJ-PMecp2-SaCas9 and AAV/DJ-gRNAi*_CREB_* viruses pseudotyped as DJ serotype. These viruses were co-infused bilaterally into the BLA, each at a titer of 1.0E13 GC/mL (1 μL/ side). Half of the animals were placed on a diet of 1 g/kg of Dox. Fourteen days later, the animals were sacrificed and their BLAs were microdissected (Figure 6B). We observed that animals receiving AAV/DJ-PMecp2-SaCas9 and AAV/DJ-gRNA*_CREB_* exhibited an editing efficiency of 28.8% [*t*(5) = 3.347, *p* = 0.0204]. Animals receiving AAV/DJ-PMecp2-SaCas9 and AAV/DJ-gRNAi*_CREB_* exhibited an editing efficiency of 25.2% when the animals were supplied Dox and virtually no editing when Dox was not present [*t*(5) = 3.347, *p* = 0.0204] (Figures 6C,D). There was no significant difference between the constitutively active and Dox-inducible systems [*t*(10) = 0.477, *p* = 0.643]. Genome editing percentages were averaged from six independent samples per group.

### AAV SaCas9-DIO CRISPR Systems Exhibit Cre-Dependent Editing *in vivo*

**FIGURE 7:**
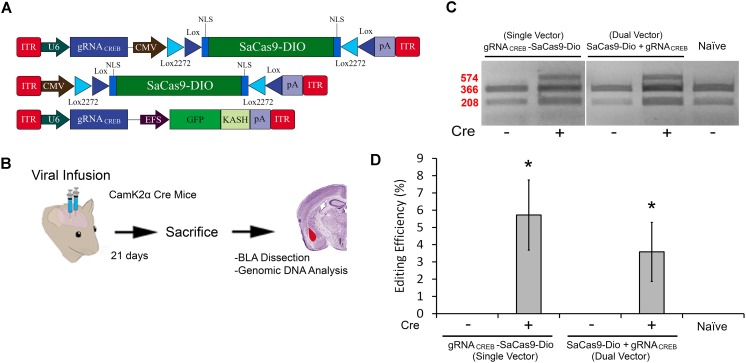
**(A)** AAV-mediated floxed SaCas9/CRISPR systems exhibit Cre-dependent editing *in vivo*. **(A)** AAV vector maps depicting CMV-SaCas9-DIO, U6-gRNA*_CREB_*-CMV-SaCas9-DIO, and U6-gRNA*_CREB_*-GFP-KASH. **(B)** Timeline from viral infusion of the virus into BLA of CaMKIIα mice, to BLA dissection, genomic DNA isolation and genome editing analysis *via* RFLP. Mice were infused with AAV/DJ-P*_CMV_*-SaCas9-DIO and AAV/DJ-gRNA*_CREB_*, or AAV/DJ-gRNA_CREB-_P*_CMV_*-SaCas9-Dio **(C)** Gel showing processed samples. Samples in lanes 1–2 received the single vector Cre-dependent system U6-gRNA*_CREB_*-CMV-SaCas9-DIO, and samples in lanes 3–4 received the dual vector Cre-dependent system AAV/DJ-P*_CMV_*-SaCas9-Dio and AAV/DJ-gRNA*_CREB_*. The fifth lane is from tissue processed alongside these samples that did not receive any viral infusion. Similar results were obtained in six independent samples per group. **(D)** Genome editing quantification. BLA samples transduced with the single vector system, AAV/DJ-gRNA_CREB-_P*_CMV_*-SaCas9-Dio, exhibited an average editing efficiency of 5.8%, while samples transduced with the dual vector system, AAV/DJ-P*_CMV_*-SaCas9-Dio and AAV/DJ-gRNA*_CREB_*, exhibited an average editing efficiency of 3.6% [*H*(2, *N* = 24) = 16.819, *p* = 0.0002, Kruskal–Wallis]. Differences between the two groups failed to reach significance [*t*(14) = 0.926, *p* = 0.3701].^∗^*p* < 0.05, compared to naive, error bars = standard error of the mean.

In our final experiment, we wanted to test our single and dual vector Cre-dependent SaCas9 systems *in vivo* (Figure 7A). AAV/DJ-Pcmv-SaCas9-DIO and AAV/DJ-gRNA*_CREB_*, or AAV/DJ-gRNA*_CREB_*-Pcmv-SaCas9-DIO viruses were produced and pseudotyped as the DJ serotype. We tested these systems using a CaMKIIα transgenic mouse line that expresses Cre-recombinase in excitatory neurons in the forebrain and, particularly, in the BLA. Our negative controls were wildtype C57BL/6 mice which also received the single and dual viral combinations. For the dual vector system, the viruses were co-infused bilaterally into the BLA, each at a titer of 4.0E12 GC/mL (1 μL/ side). The single vector system was infused bilaterally into the BLA at a titer of 8.0E12 GC/mL (1 μL/ side). Twenty-one days later, the animals were sacrificed and their BLAs were microdissected (Figure 7B). No editing was observed in the wildtype (C57BL/6) mice, indicated by “-.” Editing was observed in the Cre expressing CaMKIIα mice in both AAV/DJ-Pcmv-SaCas9-DIO and AAV/DJ-gRNA*_CREB_*, or AAV/DJ-gRNA*_CREB_*-Pcmv-SaCas9-DIO (Figure 7C). Similar results were obtained in 6 independent samples per group. The editing efficiency of the single vector system was 5.8% and the editing efficiency of the dual vector system was 3.6% [*H*(2, *N* = 24) = 16.819, *p* = 0.0002, Kruskal–Wallis]. The editing difference between these two systems did not reach significance [*t*(14) = 0.9260, *p* = 0.3701] (Figure 7D).

## Discussion

Here, we report the development of two SaCas9 systems that can be delivered to cells *via* AAV: a Cre-recombinase regulated and a Dox-inducible system. The Dox-inducible system is an extension of our previously developed Dox regulated SpCas9 system (de Solis et al., 2016). We demonstrate that both of our systems can be used to efficiently edit genomic DNA *in vitro* and *in vivo.* We also developed single and dual vector systems regulated by Cre-recombinase. The single vector system is advantageous because a single virus contains both the Cas9 and gRNA. Therefore, only one virus is needed to mediate genome editing in Cre animals or Cre cell lines. Both our systems proved to be tightly regulated with no editing shown in the absence of Dox or Cre. We think these systems will be extremely useful and can be used separately or in combination for experiments requiring tight spatial and temporal control.

In general, we observed some differences in genome editing across our experiments. For example, the standard non-inducible system achieved genomic editing of ∼30–60% when targeting 293FT and N2A cells *in vitro*. By contrast, our Dox-inducible system exhibited somewhat less efficient editing *in vitro* (∼15% less efficient genome editing compared to the non-inducible system). By contrast, in our *in vivo* experiments, we did not observe significant differences in genome editing between these systems, where both achieved approximately ∼25% genome editing. These editing efficiencies are similar to a previous study from our laboratory examining SpCas9 genome editing (de Solis et al., 2016). We suspect the Dox-inducible system may be slightly less efficient at editing *in vitro* due to lower gRNA expression from the H1 or U6 hybrid TO promoters, but we have no data to support this. But it is a reasonable possibility given that the success of the Dox-inducible systems are dependent on maintaining tight levels of control over gRNA expression. Furthermore, when the Dox-inducible systems are given more time to edit their target locus as is the case in our *in vivo* experiments, we do not see differences in editing efficiency between these systems.

For *in vivo* genome editing of neurons within the mouse brain, the average amount of genomic editing was ∼ 25%. This is likely due to a variety of reasons. In our experiments, to detect genome editing *in vivo*, a small 500-μm circular punch was taken from a 200-μm coronal section containing the amygdala that was transduced with AAV. Our AAV-SaCas9 viruses targeted neurons due to the natural tropism of AAV for neurons within the mouse brain and the fact that SaCas9 expression was controlled by an Mecp2 promoter (Swiech et al., 2015). Since ∼50% of the cells in this tissue would be glia, the highest amount of editing one would expect would be 50%. Also, these types of approaches will not transduce 100% of neurons within the microdissected tissue punch, so we typically see *in vivo* editing utilizing this approach to be ∼20–25%.

Our Cre-dependent genome editing system performed well *in vitro*, similar to the non-inducible system. However *in vivo*, the Cre-dependent system appeared to edit at a much lower efficiency ∼ 5%. In our experiments, we infused our viruses into the BLA of CamKII-Cre mice. Therefore, genome editing will be restricted to CamKII-positive excitatory neurons within this region. On average the BLA contains probably 50–60% excitatory neurons and 40–50% inhibitory neurons. So in these experiments, editing would likely be about half of what we see in our non-inducible system, which would have been ∼10–12%. Finally, the ability for Cre to flip the SaCas9 DIO transgene within AAV likely does not occur with 100% efficiency thus further reducing the editing efficiency.

Editing for all of the systems likely could be optimized further, by utilizing highly efficient gRNAs, high titer AAVs, increasing the incubation time of these viruses *in vivo*, and utilizing serotypes of AAV that transduce the target cells with high efficiency. In our single vector Cre system, we utilized a viral titer of 8.0E12 GC/mL (1 μL/ side), which is a fairly high dose. It is possible, however, to obtain titers above this and, therefore, increasing the viral titer to 3.30E13 GC/mL (1 μL/side) might boost the editing efficiency further. We utilized the DJ serotype of AAV which we have shown to transduce amygdala neurons with fairly high efficiency. However, other serotypes such as AAVDJ/8, AAV9, or AAV7 might provide better *in vivo* transduction (Holehonnur et al., 2014). Finally, we infused our Cre-dependent AAVs and harvested the tissue 3 weeks later for genome editing analysis. If we harvested at 4 or 5 weeks, we may have been able to detect more genome editing.

Several CRISPR/Cas9-based-inducible systems have been developed, but none have been developed for the SaCas9 variant. Since the characterization of SaCas9 (Ran et al., 2015a), several groups have extended its application in targeted mutagenesis in a variety of models such as plants, mice, and zebrafish (Steinert et al., 2015; Feng et al., 2016; Zhang et al., 2016; Kaya et al., 2017). Due to its smaller size, SaCas9 has substantial advantages in delivery and expression of Cas9, especially when using AAVs. Recent work has shown that SaCas9 possesses higher activity than the other Cas9 variants such as SpCas9 and FnCpf1 (Xie et al., 2017). Furthermore, a recent study demonstrates a split SaCas9 system as a genome editing tool in plants (Kaya et al., 2017). They show that SaCas9 can be split, and that the split-SaCas9 expressed from *Agrobacterium* can induce targeted mutagenesis in *Nicotiana benthamiana.* This split-SaCas9 has almost the same activity as that of full-length SaCas9, providing us with the smallest tool yet for CRISPR.

To our knowledge, there are currently no available Cre-dependent or Dox-inducible SaCas9 vector systems that have been validated. These systems not only provide comparable editing levels to our previously developed Dox-inducible SpCas9 system but also have the advantage of being within the AAV packaging limit. Moreover, these systems can likely be used in combination to provide both spatial and temporal control of viral-mediated genome editing. For example, utilizing our two systems in conjunction could enable researchers in developmental neuroscience to restrict genome editing to targeted regions *via* the Cre-dependent system and an appropriate Cre mouse line, while inducing genome editing at desired time points *via* the Dox-inducible system. This greatly facilitates the investigation of the developmental role of any targetable gene without disrupting the gene’s expression in surrounding regions. Similar approaches can be applied to behavioral neuroscience. For example, investigators can benefit from the ability to selectively manipulate gene expression with precise spatial and temporal resolution.

## Author Contributions

NK, WS, NA, and T-MD engineered the viral plasmids. NK, WS, and CdS performed the ICC. NK, WS, A, NA, T-MD, ST, and AS tested the efficacy of editing of viral plasmids *in vitro*. NK and CdS produced, purified, and titered the viruses. CdS performed all viral infusions and microdissection. WS and CdS examined efficiency of editing *in vivo*. CG produced and maintained the CaMKIIα-Cre transgenic mice for the *in vivo* Cre-dependent system. NK, WS, CdS, and JP conceived the study and participated in its design and coordination and drafted the manuscript. All authors read, edited, and approved the final manuscript.

## Conflict of Interest Statement

The authors declare that the research was conducted in the absence of any commercial or financial relationships that could be construed as a potential conflict of interest.

## Footnotes

1 http://imagej.nih.gov/ij/
